# Supervised Exercise Interventions in Childhood Cancer Survivors: A Systematic Review and Meta-Analysis of Randomized Controlled Trials

**DOI:** 10.3390/children9060824

**Published:** 2022-06-02

**Authors:** Qing Shi, Junyi Zheng, Ke Liu

**Affiliations:** School of Nursing, Sun Yat-sen University, Guangzhou 510080, China; shiq6@mail2.sysu.edu.cn (Q.S.); zhengjy66@mail2.sysu.edu.cn (J.Z.)

**Keywords:** exercise, childhood cancer survivors, adherence, health outcomes

## Abstract

Background: Childhood cancer survivors (CCSs) may suffer from a multitude of health impairments, resulting in a compromised quality of life (QoL). This review’s objective was to examine CCSs’ adherence to supervised exercise training interventions and the impact of these interventions on health outcomes. Methods: The following databases were searched in May 2022: PubMed, Embase, Cochrane Library, and Web of Science. The included studies were limited to randomized controlled trials (RCTs), published in English involving CCSs aged 18 years and below. Results: Nine RCTs (*n* = 642 participants) were included in the systematic review, and seven of them (*n* = 551 participants) were included in the meta-analysis. Both the mean retention rate and adherence to the supervised exercise interventions were 87%. Supervised exercise interventions significantly improved muscle strength (standardized mean difference (SMD) = 1.42, *p* = 0.03), level of daily physical activity (SMD = 1.05, *p* < 0.001), body mass index (BMI) (mean difference (MD) = 1.06, *p* = 0.03), and fatigue (SMD = −0.44, *p* < 0.001), while there was no statistical significance in the quality of life (QoL) (SMD = 0.21, *p* = 0.20). Conclusions: The adherence of CCSs to supervised exercise interventions is high, and supervised exercise interventions are safe and effective.

## 1. Introduction

Cancer and anticancer therapies are associated with many adverse effects on childhood cancer survivors (CCSs). Research has shown that, during treatment, CCSs may experience fatigue and a decline in cardiopulmonary function, muscle strength, functional performance, and quality of life (QoL) [1,2,3]. Although, the five-year survival rate of children diagnosed with cancer has reached nearly 85% due to the significant advancements in cancer treatment [4]. CCSs face a high risk of severe and even fatal late health consequences of cancer or treatment [5]. It is estimated that approximately two-thirds of survivors may experience at least one complication, such as obesity, diabetes, osteoporosis, cardiovascular disease, and secondary malignant tumors [5,6,7,8]. These adverse effects can have a negative impact on CCSs’ health outcomes.

As an effective nonpharmacological therapy, exercise plays an essential role in the treatment of CCSs, which can improve motor function ability and exercise tolerance [9,10]. However, up to 60~75% of CCSs’ level of daily physical activity does not meet the World Health Organization’s (WHO) recommended average of 60 min per day of at least moderate-intensity physical activity [11,12,13]. Physical inactivity has been identified as an important reason for diminished physical function in CCSs [14]. Previous meta-analyses of CCSs found that exercise interventions can reduce the side effects of cancer treatment in CCSs and bring health benefits [14,15]. In particular, supervised exercise is one of the most effective exercise modalities representing modifiable health behavior. The implementation of a supervised exercise intervention can reduce cancer-related sequelae (e.g., fatigue) and improve the mental health and QoL of adult cancer survivors [16]. QoL refers to the experience of living conditions from the subjective perspective of individuals in different cultures and value systems [17]. However, evidence shows low adherence to physical activity among adult cancer survivors [18]. It has been found that supervision acts as a valuable add-on in improving the adoption of and adherence to an exercise intervention [19,20]. Adherence to exercise programs is an essential component of preventing and managing chronic health conditions, such as cancer. The supervising health professionals can carry out personalized exercises according to the specific situation of each person and can provide timely encouragement and help [21]. In addition, due to children’s physical and mental immaturity, supervision makes CCSs more motivated and confident in performing the exercise.

At present, three previous systematic reviews and meta-analyses have addressed the effect of exercise interventions in CCSs [14,15,22]. In the existing three reviews, Braam’s [14] research mainly included homogeneous childhood acute lymphoblastic leukemia patients, which was diagnosis-specific. Moreover, two of these studies [14,15] were delivered during treatment for CCSs, and the other one included an unsupervised remote exercise intervention [22]. The findings from these previous systematic reviews and meta-analyses cannot be generalized to CCSs, owing to the differences in disease diagnosis, prognosis, and exercise intervention delivery, as well as between children undergoing active cancer treatment and children having completed treatment. However, evidence-based outcomes of supervised exercise interventions are focused on adult cancer survivors [23]. Furthermore, evidence supporting the effect of supervised exercise interventions is lacking in CCSs. Therefore, in view of this gap in the literature and the value of supervised exercise interventions, we sought to address two key questions to further understand the knowledge of exercise-oncology in CCSs: (1) What are the retention, adherence rate, and safety of exercise interventions during and after treatment? and (2) What is the effect of exercise interventions on physical activity levels and health outcomes in CCSs?

## 2. Materials and Methods

This study was conducted according to the Preferred Reporting Items for Systematic Reviews and Meta-Analysis (PRISMA) [24] and the Cochrane Collaboration Handbook [25]. The protocol’s PROSPERO registration number is CRD42020220480.

### 2.1. Eligibility Criteria

We included studies that met the following criteria: (1) participants: children aged 18 years and below with a cancer diagnosis (during or after treatment); (2) intervention: all exercise interventions were supervised by health professionals, medical staff, or coaches and included aerobic, anaerobic, resistance, or combined physical exercise training regimens; (3) comparisons: usual care or placebo intervention; and (4) design: randomized controlled trial (RCT).

Studies of telephone monitoring, activity monitoring, and manual therapy were excluded, because the supervision function of telephone and activity monitoring is weak [26], and manual therapy is mainly composed of passive movements, such as massage, bone rectification, and mobilization.

### 2.2. Search Strategy

Searches were carried out using PubMed, Embase, Cochrane Library, and Web of Science in May 2022. The search strategy was based on synonyms and Medical Subject Headings (MeSH) of the key concepts of cancer, children, and exercise. The following search terms were used: (cancer OR oncology OR tumor OR tumour OR neoplasm OR leukemia OR leukaemia OR carcinoma OR sarcoma OR malignant OR maligna*) AND (pediatric OR paediatric OR child OR child* OR kid OR infant OR adolescent OR adoles* OR teenager OR teen*) AND (physical activity OR exercise OR aerobic OR resistance OR training OR sport OR physical therapy OR rehabilitation). The detailed search strategy is described in Appendix A. In addition, we manually checked the references of the included articles and published systematic reviews on exercise interventions.

### 2.3. Data Extraction

Two authors (Q.S. and J.Z.) independently screened the titles and abstracts of the articles to determine eligibility. Then, full texts of potentially relevant studies were retrieved for further assessment of their eligibility. Data from the included studies were independently extracted and summarized by two authors (Q.S. and J.Z.) using a standardized data extraction form. If there was any disagreement, the third author was involved in the discussion until a consensus was reached. We extracted and summarized the following information for all of the included studies: Study design, basic information of the studies, characteristics of the participants, intervention characteristics (i.e., frequency, intensity, time, type, and setting), outcome measures (i.e., retention, adherence rates and safety, cardiorespiratory fitness, muscle strength, functional performance, flexibility, balance, level of daily physical activity, body mass index (BMI), total lean and fat mass, fatigue, QoL, and self-efficacy).

### 2.4. Quality Assessment

The quality and risk of bias of the studies were assessed according to the Cochrane Handbook for Systematic Reviews of Interventions [25]. Two authors (Q.S. and J.Z.) evaluated the following criteria: random sequence generation (selection bias), allocation concealment (selection bias), blinding of participants and personnel (performance bias), blinding of outcome assessor (detection bias), incomplete outcome data (attrition bias), selective reporting (reporting bias), and other potential sources of bias. Each domain was classified into three levels: “Low risk”, “high risk”, or “unclear”. Quality assessment was evaluated by two independent authors (Q.S. and J.Z.), and disagreements were resolved through discussion with the third author (K.L.).

### 2.5. Statistical Analysis

Review Manager 5.3 and STATA software were used to perform a meta-analysis of the included studies. The means and standard deviations (SDs) from baseline to post-intervention were recorded. For continuous outcomes, if the measuring tool was the same, we adopted the mean difference (MD) with 95% confidence intervals (95% CIs); if the measuring instruments were inconsistent, we used the standard mean difference (SMD) with 95% CIs. Statistical heterogeneity was calculated with I^2^ statistic, which was explained using the following cut-off parameters: non-important heterogeneity, 0% to 40%; moderate heterogeneity, 30% to 60%; substantial heterogeneity, 50% to 90%; and considerable heterogeneity, 75% to 100% [25]. If the heterogeneity was substantial (I^2^ > 50%), a random-effects model was applied; otherwise, a fixed-effect model was used [27]. If there were several articles based on the same study, only one study was included in the meta-analysis. When measurements were conducted at different time points, the data closest to the end of the intervention were included. If the heterogeneity was substantial (*p* < 0.1, I^2^ > 50%), sensitivity analysis was conducted by excluding studies one by one to explore the possible source of heterogeneity. Potential publication bias was evaluated by Egger’s test. A two-sided *p* < 0.05 was considered to be statistically significantly different. 

## 3. Results

### 3.1. Study Selection

**Figure 1:**
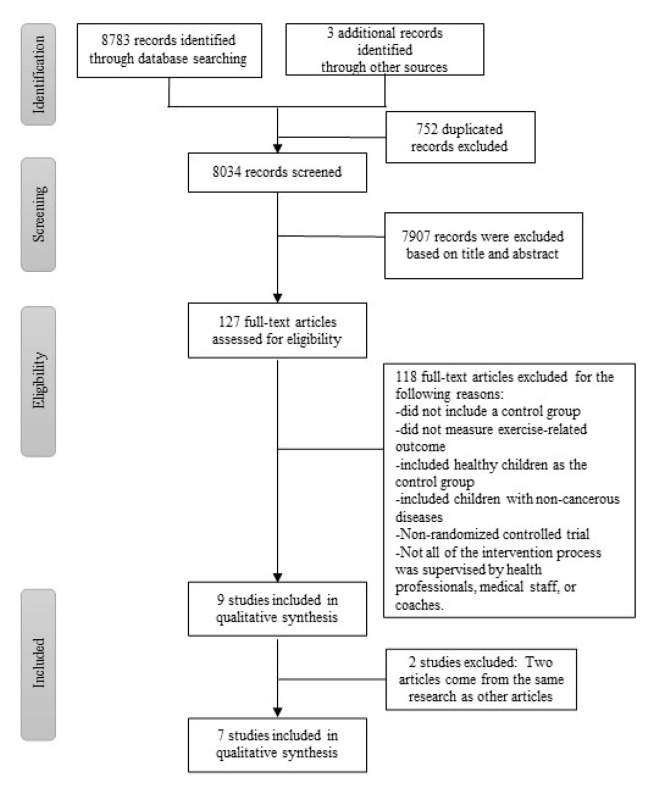
PRISMA flow chart illustrating the detail of the search strategy.

The initial search retrieved a total of 8786 records, which was reduced to 8034 studies after removing duplicates. After screening the titles and abstracts of the identified articles, 7907 articles were excluded as they did not meet the inclusion criteria. The full texts of the remaining 127 articles were reviewed; finally, nine articles were eligible for inclusion and were included in this review [28,29,30,31,32,33,34,35,36]. Four articles were published on the same two studies, with the remaining seven RCT studies for the quantitative analysis (Figure 1) [29,31,32,34,35,36].

### 3.2. Quality Assessment

**Figure 2:**
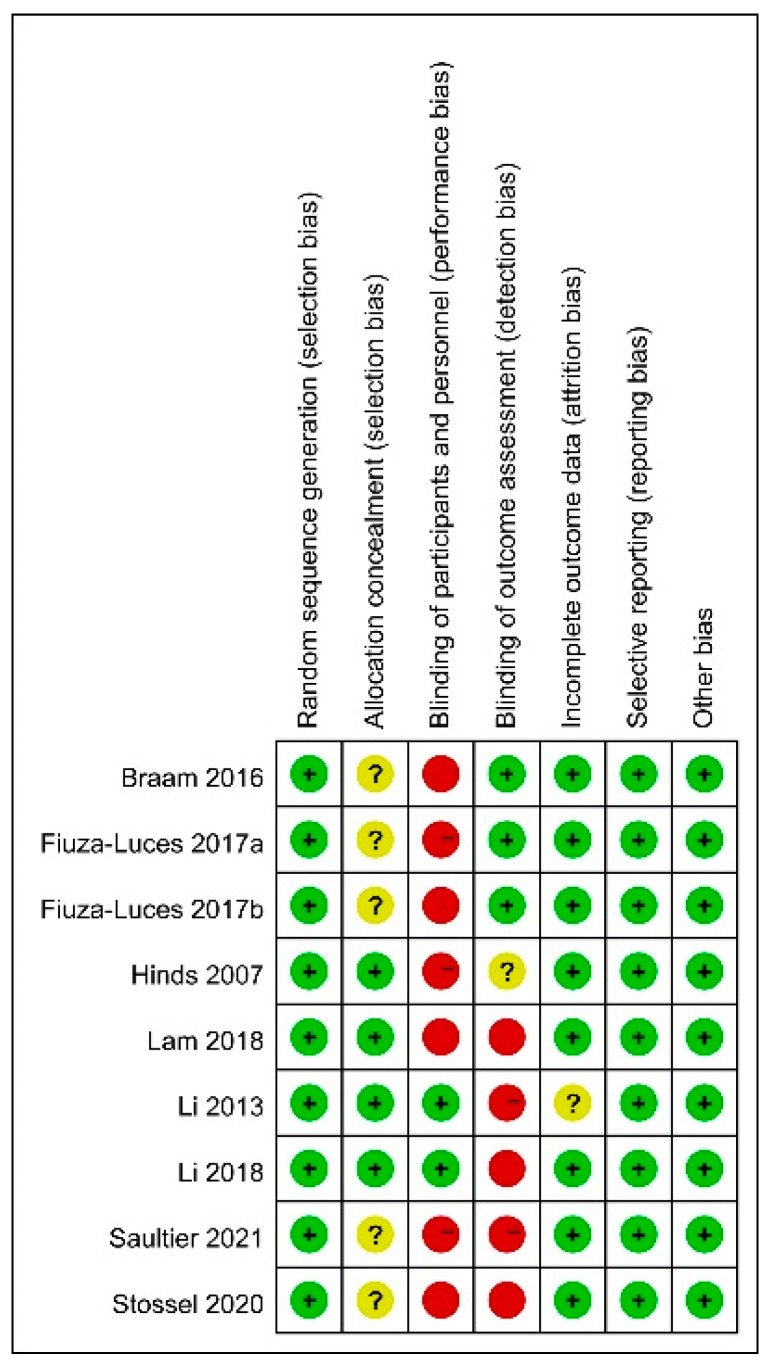
Quality of included studies.

The overall risk of bias of the included studies was moderate (Figure 2). All of the RCTs mentioned randomization and described, in detail, the method of random sequence generation [28,29,30,31,32,33,34,35,36]. It was impossible to perform a double-blind method due to the nature of the intervention. Therefore, all studies failed to use the double-blind method, leading to a high risk of bias in the blinding of participants and personnel and/or the blinding of the outcome assessment [28,29,30,31,32,33,34,35,36]. Four articles [31,32,33,34] adequately reported allocation concealment, while five [28,29,30,35,36] did not mention it.

### 3.3. Systematic Review and Meta-Analysis

#### 3.3.1. Participants’ and Intervention Characteristics

Table 1 shows the details of the included study characteristics. The sample size of the included studies varied from 20 [30] to 222 [34] participants. The total number of included participants was 642, of which 322 belonged to the experimental group. The age of the participants ranged from 4 [29] to 18.4 [35] years. All studies focused on CCSs with mixed types of cancer [28,29,30,31,32,33,34,35,36].

All studies included pre- and post-intervention evaluation [28,29,30,31,32,33,34,35,36]. Moreover, five studies also evaluated the results from follow-ups 2~18 months after the intervention [29,30,32,33,34]. Although one study combined exercise training with a psychosocial intervention [28], the rest only included an exercise intervention [29,30,31,32,33,34,35,36]. Two studies of exercise interventions were conducted after the end of treatment [33,34], and six were conducted during treatment [29,30,31,32,35,36]. The mean duration of the supervised exercise interventions was 16.60 weeks (SD = 8.13), including a mean of 2.25 sessions (SD = 1.70) per week, and the sessions lasted a mean of 152.36 min (SD = 168.84). All studies had control groups that received either the usual or placebo intervention [28,29,30,31,32,33,34,35,36]. The intervention types of six studies were resistance and aerobic interventions [28,29,30,33,34,36]; the interventions in two studies included multiple types of exercise (i.e., resistance, aerobic, and stretching training) [32,35]; and one study only included an aerobic intervention [31]. Four studies were of high intensity [29,30,35,36], and one was of medium and low intensity [32].

#### 3.3.2. Adherence to the Exercise Program

Nine studies included retention rates ranging from 70% to 100% [28,29,30,31,32,33,34,35,36]. The mean retention rate of these studies was 87%. Adherence to interventions was demonstrated in five studies, ranging from 68% to 100% [29,31,32,33,34], with a mean adherence of 87%. In general, the adherence to supervised exercise interventions was high.

#### 3.3.3. Adverse Effects

There were no adverse effects in six studies [29,30,31,33,34,35]. One study reported no serious adverse effects, but mentioned falls and muscle soreness [36].

#### 3.3.4. Health Outcomes

**Figure 3:**
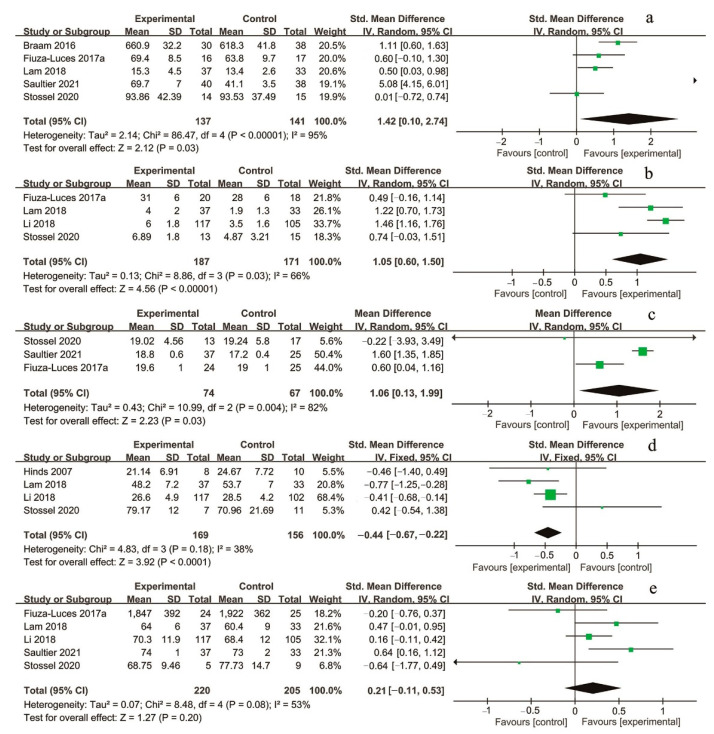
Meta-analyses of the effect of supervised exercise interventions on (**a**) muscle strength, (**b**) level of daily physical activity, (**c**) BMI, (**d**) fatigue, and (**e**) QoL.

**Figure 4:**
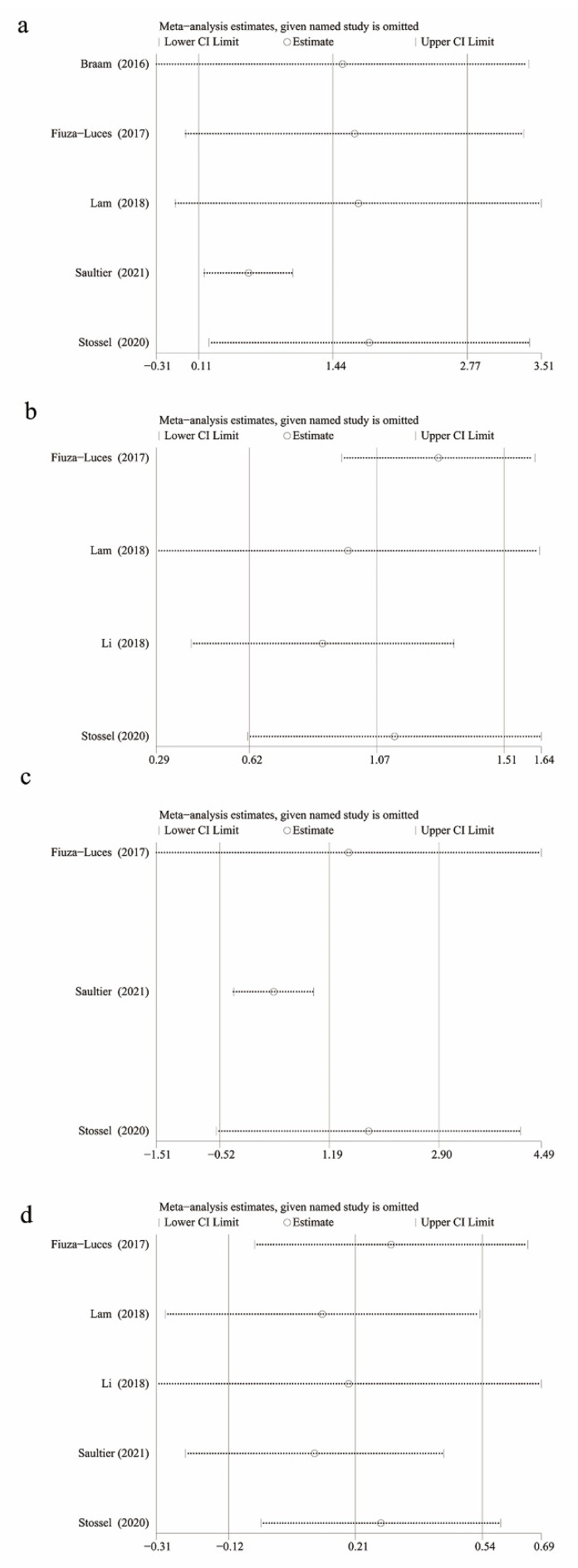
Sensitivity analysis of (**a**) muscle strength, (**b**) level of daily physical activity, (**c**) BMI, and (**d**) QoL.

1. *Cardiorespiratory fitness*: Four studies analyzed the effects of an exercise intervention on cardiorespiratory fitness [28,29,35,36]. Cardiorespiratory fitness was evaluated by VO_2peak_ [28,29], ventilatory threshold [28], and the six-minute walk test [35,36]. Two studies found improvements in cardiopulmonary fitness after the exercise interventions [35,36].
2. *Muscle strength*: Five studies evaluated the effect of an exercise intervention on muscle strength [28,29,32,35,36]. Three of them were measured with handheld dynamometers (the highest of the three repetitions was counted as the maximum strength) [28,32,36]; one [29] examined dynamic upper and lower body muscle strength endurance using five repeat maxima of bench, row, and leg press machines [37]; and another study [35] reported upper limb strength (1 kg medicine ball launch), lower limb strength (Myotest^®^ [38] and chair test) [39], trunk muscle endurance (bridge trunk muscle endurance test), and abdominal muscle endurance (sit-up score) [40]. Four studies found that exercise interventions can significantly enhance muscle strength [29,32,35,36].
  According to the meta-analysis results, muscle strength can significantly improve in CCSs who receive a supervised exercise intervention compared with the control group (*n* = 5 studies, *n* = 300 participants, SMD = 1.42, 95% CI = 0.10~2.74, *p* = 0.03) [28,29,32,35,36]. There was considerable heterogeneity (I^2^ = 95%, *p* < 0.001) (Figure 3a). The sensitivity analysis did not identify any single study affecting the overall results more than other studies (Figure 4a).
3. *Functional performance*: Only one study analyzed the effect of the exercise intervention on functional performance, using the 3 m Timed Up and Go (TUG) test and Timed Up and Down Stairs (TUDS) test [29]. This study did not report a beneficial training effect of the exercise intervention on functional performance [29].
4. *Flexibility and balance*: One study assessed the effect of the exercise intervention on flexibility and balance [35]. Flexibility and balance were measured by the sit and reach test and flamingo balance test, respectively. The results showed that the program can effectively improve the flexibility and balance of CCSs.
5. *Level of daily physical activity*: Six studies analyzed the effect of exercise interventions on the level of daily physical activity [29,30,32,33,34,36]. These studies adopted the Chinese University of Hong Kong Physical Activity Rating for Children and Youth scales [32,33,34], the German Momo questionnaire [36], or acceleration for objective measurement [29,30]. Three studies found that the level of daily physical activity increased after exercise interventions [32,33,34].
  Based on the meta-analysis, compared to the control group, supervised exercise can significantly increase the level of daily activity of CCSs in the experimental group (*n* = 4 studies, *n* = 374 participants, SMD = 1.05, 95% CI = 0.60~1.50, *p* < 0.001), with substantial heterogeneity between studies (I^2^ = 66%, *p* = 0.03) (Figure 3b) [29,32,34,36]. The results of the sensitivity analysis demonstrated that the removal of any studies had no significant effect on the overall results, indicating that this meta-analysis is robust (Figure 4b).
6. *Body composition*: Three studies assessed BMI. [29,35,36]. One study used an impedance meter to measure the total lean and fat mass [36]. No studies found a significant effect on body composition [29,35,36].
  The meta-analysis showed that, compared to the control group, supervised exercise can significantly increase CCSs’ BMI in the experimental group (*n* = 3 studies, *n* = 162 participants, MD = 1.06, 95% CI = 0.13~1.99, *p* = 0.03) [29,35,36]. Substantial heterogeneity existed among the three studies (I^2^ = 82%, *p* = 0.004) (Figure 3c). Sensitivity analysis confirmed that the results of BMI are robust and reliable (Figure 4c).
7. *Fatigue*: Four studies analyzed the effects of exercise training on fatigue [31,32,34,36]. The Fatigue Scale was employed in three studies [31,32,34]; in one of the studies, children, adolescents, parents, and medical staff all provided reports of fatigue [31]. Another study used the Pediatric QoL Inventory 3.0 (PedsQL 3.0) multidimensional fatigue scale. Three studies found that exercise interventions can decrease fatigue [32,34,36].
  Our meta-analysis demonstrated that supervised exercise interventions can significantly reduce fatigue in the experimental group compared to the control group (*n* = 4 studies, *n* = 354 participants, SMD = −0.44, 95% CI = −0.67~−0.22, *p* < 0.001), and there was not important heterogeneity in fatigue (I^2^ = 38%, *p* = 0.18) (Figure 3d) [31,32,34,36].
8. *QoL*: Six studies assessed the impact of exercise interventions on QoL [29,32,33,34,35,36]. Two studies used PedsQL 3.0 [29,32]; two studies employed version 4.0 of this questionnaire [33,34]; one study applied the German language KINDL questionnaire [36]; and one study adopted the “Vécu et Santé Perçue de l’Adolescent et de l’enfant” questionnaire (VSP-A) [35]. It was found that exercise interventions improved the QoL in three studies [33,34,35].
  Compared to the control group, supervised exercise interventions did not significantly improve the QoL of CCSs in the experimental group (*n* = 5 studies, *n* = 454 participants, SMD = 0.21, 95% CI = −0.11~0.53, *p* = 0.20). Moderate heterogeneity was found between studies (I^2^ = 53%, *p* = 0.08) (Figure 3e) [29,32,34,35,36]. Sensitivity analysis suggested no significant effect on the overall results by omitting any studies (Figure 4d).
9. *Self-efficacy*: Three studies adopted the Physical Activity Self-Efficacy scale (PA-SE) to evaluate the effect of the exercise interventions on self-efficacy, the results of which showed that exercise interventions had a significant effect on self-efficacy [32,33,34].

### 3.4. Publication Bias

Overall, there was no evidence of publication bias in muscle strength (Egger’s test, *p* = 0.37), level of daily activity (Egger’s test, *p* = 0.25), BMI (Egger’s test, *p* = 0.73), fatigue (Egger’s test, *p* = 0.87), and QoL (Egger’s test, *p* = 0.75).

## 4. Discussion

To the best of our knowledge, this is the first meta-analysis to quantitatively summarize the effects of supervised exercise interventions on CCSs. This systematic review and meta-analysis provides new evidence for the effect of supervised exercise interventions on CCSs. The results demonstrated that supervised exercise interventions had high retention and adherence rates, which could significantly improve muscle strength, level of daily physical activity and BMI, and reduce the fatigue of CCSs during and after treatment. In addition, no major adverse events or health-related problems related to exercise training were found. Therefore, supervised exercise interventions performed during and after treatment are safe and effective.

In this meta-analysis, substantial or considerable heterogeneity was observed in the included studies. Firstly, there were some differences in the study population. Although all of the participants were CCSs, they were in different stages of treatment when the supervised exercise interventions were implemented. Two studies were conducted when the CCSs had completed cancer treatment [33,34]; one study was in treatment or within the first year after cancer treatment [28]; and six studies were undergoing treatment, but the specific stage was not clear [29,30,31,32,35,36]. Secondly, notable differences were present in the supervised exercise interventions. The interventions in nine studies differed widely with regard to the types of exercise interventions (aerobic [31], aerobic and resistance [28,29,30,33,34,36], or multitype exercise [32,35]), duration (two days [31] to 24 weeks [32,33,34,35]), and number of sessions (three [33,34] to 57 sessions [29]). Finally, most of the outcome measures were different. For example, six measurement methods were used to measure muscle strength in this meta-analysis. However, due to the limited number of included studies, subgroup analysis could not be performed to analyze the definite source of heterogeneity in some of the outcomes.

Through supervised exercise interventions, health professionals can make exercise plans according to the current physical condition of CCSs and can provide timely feedback. Such feedback includes suggestions on the type, intensity, frequency, and duration of exercise and encouraging CSSs to exercise in their daily life [34]. This feedback can improve motivation to perform the exercise, resulting in increased adherence [41]. Previous studies have shown that the adherence to supervised exercise interventions was higher than that of home-based exercise interventions [42,43]. Supervised training programs had greater adherence among adolescents and increased training-induced adaptations than those unsupervised ones [44]. In this systematic review, the retention rate of supervised exercise interventions (87%) was slightly higher than that of non-supervised ones (85%), and adherence to supervised exercise interventions (87%) was similar to that with no supervision (88%) [22]. Unlike previous evidence [45], it was found that supervised exercise interventions in survivors do not lead to higher adherence than unsupervised exercise interventions. This may be because most of the participants in this systematic review were in their treatment period, and they were absent due to poor physical condition [46], while all of the participants in Mizrahi’s [22] systematic review had completed their intensive cancer treatment regimens. Nevertheless, this review demonstrated a positive impact of supervised exercise interventions on CCSs, and the impact still existed after the interventions [32,33,34]. Therefore, CCSs benefited more from supervised exercise interventions than unsupervised or home-based interventions. 

Cancer and its treatment result in impaired physical function in children [47]. Concretely, the muscle strength of CCSs is impaired to different degrees during and after treatment [48]. Muscle strength is indispensable in many daily-life activities for individuals to dress, walk, stand, climb stairs, etc. [49]. Lanfranconi [50] found that exercise can increase the arm and leg muscle strength of CCSs. However, there is no previous evidence specifically reporting the effects of a supervised exercise intervention on muscle strength in CCSs. Our meta-analysis demonstrated that supervised exercise interventions could significantly improve muscle strength in CCSs, both during and after treatment, which is consistent with the effect of exercise interventions in adult cancer patients [51]. Importantly, this systematic review and meta-analysis adds some details for exercise interventions modality and evidence of supervised exercise interventions on the muscle strength of CCSs.

The treatment of cancer and its sequelae can significantly reduce physical activity and increase the fatigue of CCSs [52,53]. Fortunately, supervised exercise interventions can significantly increase the level of daily physical activity and improve fatigue for CCSs, as demonstrated in this review. In contrast, distance-delivered interventions had no significant effect on the level of daily physical activity among CCSs [22]. There has been no previous meta-analysis that has evidenced that exercise interventions can improve the level of daily physical activity and fatigue in CCSs [14]. This means that greater exercise practice is driven by supervised exercise interventions. Moreover, this review also confirmed that supervised exercise interventions are one of the most effective nonpharmacological strategies for improving fatigue. This meta-analysis included Li’s study; however, the intervention frequency of Li’s study was low and the sample size was large, which may have a great impact on the meta-analysis results. Therefore, further RCTs of high-quality and reasonable intervention programs are needed to strengthen this evidence and encourage supervised exercise interventions for CCSs.

Although the results of these three studies showed no significant effect on BMI [29,35,36], our pooled meta-analysis demonstrated that supervised exercise interventions increase BMI. In three studies [29,35,36], CCSs had a relatively low BMI during treatment. Malnutrition is considered an important predictor of decreased overall survival [54]. Ouyang [55] found that 55.8% of children with cancer were malnourished, and 74.2% had a moderate-to-high risk of malnutrition. The increase in BMI suggested that supervised exercise might indirectly improve the body weight and nutritional status of CCSs. Duan [56] also found that exercise can promote BMI in adult cancer patients. Therefore, supervised interventions can be considered in daily practice to improve BMI.

The results of this meta-analysis showed no significant improvement in QoL. However, significant improvements in physical outcomes from individual RCTs, including cardiopulmonary fitness [35,36], muscle strength [29,32,35,36], flexibility and balance [35], level of daily physical activity [32,33,34], and fatigue [32,34,36], may be considered important factors in improving the QoL. Consistent with our results, the studies of the current meta-analysis did not find a significant effect of exercise on the QoL of CCSs [14,15,22]. Another study showed significant beneficial effects on QoL in adult cancer survivors through supervised exercise interventions, but not through unsupervised interventions [43]. Given the limited number of studies included in the analysis, more data from high-quality RCTs are required to derive stronger evidence on the effect of supervised exercise on QoL in CCSs.

There were several limitations in our review. This meta-analysis lacked evidence of some important outcome measures. The original meta-analysis program intended to include anxiety and depression as outcome variables. However, the included studies did not assess anxiety or depression. As a result, this review lacked evidence of the psychological impact of supervised exercise interventions. Moreover, the methodological differences between RCTs introduced a moderate risk of bias, including that some trials did not blind subjects, interveners, and/or outcome evaluators. Furthermore, the statistical heterogeneity of our results might impact the ability to draw strong conclusions from the effects of the supervised exercise interventions. Although each study used a supervised exercise program, the great variability in the outcome measures and the intervention dose may be the cause of the heterogeneity. Due to the high variability and limited studies included in the meta-analysis, subgroup analysis could not be conducted to obtain optimum results of the type, intensity, frequency, and duration of supervised exercise interventions.

## 5. Conclusions

In summary, the adherence of CCSs to supervised exercise interventions was high. Supervised exercise interventions were able to improve muscle strength, the level of daily physical activity, BMI, and fatigue in CCSs. However, supervised exercise interventions did not achieve a statistically significant level to improve QoL. More high-quality RCTs are needed to further explore the optimal type, intensity, frequency, and duration of supervised exercise interventions for CCSs and to determine their impact on psychological outcomes. Nevertheless, this evidence indicates that supervised exercise interventions are safe and effective intervention strategies for CCSs. Therefore, we recommend that supervised exercise programs be implemented to improve the physical condition of CCSs during and after treatment.

## Figures and Tables

**Table 1:** Main characteristics of supervised exercise interventions for childhood cancer survivors.

Study, Year	Sample Size	Age (Years) (Mean ± SD)	Cancer Type	Timing of the Study	Intervention	Retention Rate and Adherence	Adverse Effects	Endpoints	Main Findings
Braam, et al., 2017 [26]	*N* = 68 EG: (*n* = 30) CG: (*n* = 38)	EG: 13.4 ± 3.1 CG: 13.1 ± 3.1	Mixed cancer	During cancer treatment or within the first year after cancer treatment	EG: *Frequency*: 2 days/week *Intensity*: NR *Time*: 12 weeks *Type*: resistance and aerobic training *Settings*: physical therapy center CG: Usual care	Retention: 86.7% Adherence: NR	NR	**VO_2peak_** (the cardiopulmonary exercise test) **Muscle strength** (handheld dynamometer) **QALYs** (EQ-5D-Y, PedsQL™) **Cost** (cost questionnaires, the mean hourly productivity cost of the Dutch population)	-No major training effect
Fiuza-Luces, et al., 2017a [27]	*N* = 49 EG: (*n* = 24) CG: (*n* = 25)	EG: 10 ± 1 CG: 11 ± 1	Mixed cancer	During treatment (treatment stage include the entire neoadjuvant chemotherapy treatment period)	EG: *Frequency*: 3 days/week *Intensity*: aerobic training: 60~70% of maximum heart rate & resistance training: N/R *Time*: 17 ± 5 weeks *Type*: resistance and aerobic training *Settings*: hospital CG: Usual care	Retention: 100% Adherence: 68% ± 4%	No	**Muscle strength** (5-RM seated bench, row, and leg press machines) **VO_2peak_** (breath-by-breath, arm crank ergometer test) **Ventilatory threshold** (breath-by-breath, arm crank ergometer test) **BMI** **Functional capacity** (TUG, TUDS) **PA** (accelerometer) **QoL** (PedsQL Cancer Module 3.0)	-↑Muscle strength
Fiuza-Luces, et al., 2017b [28]	*N* = 20 EG: (*n* = 9) CG: (*n* = 11)	EG: 11 ± 4 CG: 12 ± 4	Mixed cancer	During treatment (treatment stage include the entire neoadjuvant chemotherapy treatment period)	EG: *Frequency*: 3 days/week *Intensity*: aerobic training: 60~70% of maximum heart rate & resistance training: N/R *Time*: 17 ± 5 weeks *Type*: resistance and aerobic training *Settings*: hospital CG: Usual care	Retention: 70% ± 13% Adherence: NR	No	**Immune function** (blood samples) **Inflammation markers** (blood samples) **PA** (accelerometer)	-No major training effect
Hinds, et al., 2007 [29]	*N* = 29 EG: (*n* = 14) CG: (*n* = 15)	EG: 13.1 ± 2.6 CG: 11.9 ± 3.2	Mixed cancer	During treatment	EG: *Frequency*: 2 times/day *Intensity*: N/R *Time*: 2~4 day *Type*: aerobic training *Settings*: hospital CG: Usual care	Retention: 85.37% Adherence: 100%	No	**Sleep efficiency** and sleep duration (wrist actigraph, DSDP) **Fatigue** (FS-C, FS-A, FS-P, FS-S) **Hemoglobin** (blood samples) **Hematocrit** (blood samples)	-↑Sleep efficiency
Lam et al., 2018 [30]	*N* = 70 EG: (*n* = 37) CG: (*n* = 33)	EG: 12.8 ± 2.5 CG: 12.5 ± 2.5	Mixed cancer	During treatment (treatment stage not specified)	EG: *Frequency*: 2 days/week for the first 4 weeks, and then 1 day/weeks for 20 weeks *Intensity*: low and moderate *Time*: 24 weeks *Type*: stretching, relaxation exercises, strengthening and resistance exercises, and aerobic exercises *Settings*: home and hospital CG: Placebo intervention	Retention: 91.9% Adherence: 89.2%	NR	**Fatigue** (FS-C) **QoL** (PedsQL Cancer Module 3.0) **PA** (CUHK-PARCY) **Right- and left-hand grip strength** (Handheld dynamometers) **Self-efficacy** (PA-SE)	-↓Fatigue -↑QoL -↑PA -↑Right-hand grip strength -↑Left-hand grip strength -↑Self-efficacy
Li, et al., 2013 [31]	*N* = 71 EG: (*n* = 34) CG: (*n* = 37)	EG: 12.5 ± 2.2 CG: 12.8 ± 2.1	Mixed cancer	At least 6 months after completing cancer treatment	EG: *Frequency*: 4 days at 2 weeks and 2, 4, and 6 months after randomization Intensity: N/R *Time*: 6 months *Type*: resistance and aerobic training *Settings*: camp training center CG: Placebo intervention	Retention: 91.2% Adherence: 85.3%	No	**QoL** (PedsQL) **PA** (CUHK-PARCY) **Self-efficacy** (PA-SE) **Physical activity stages of change** (PASCQ)	-↑PA -↑Self-efficacy -↑Physical activity stages of change
Li, et al., 2018 [32]	*N* = 222 EG: (*n* = 117) CG: (*n* = 105)	EG: 12.8 ± 1.9 CG: 12.5 ± 2.6	Mixed cancer	At least 6 months after completing cancer treatment	EG: *Frequency*: 4 days at 2 weeks and 2, 4, and 6 months after randomization *Intensity*: N/R *Time*: 6 months *Type*: resistance and aerobic training *Settings*: camp training center CG: Placebo intervention	Retention: 88.0% Adherence: 91.5%	No	**Fatigue** (FS-C) **QoL** (PedsQL 4.0) **PA** (CUHK-PARCY) **Self-efficacy** (PA-SE)	-↓Fatigue -↑QoL -↑PA -↑Self-efficacy
Saultier, et al., 2021 [33]	*N* = 80 EG: (*n* = 41) CG: (*n* = 39)	EG: 11.4 ± 0.6 CG: 11.2 ± 0.6	Mixed cancer	During treatment	EG: *Frequency*: 2 days/week *Intensity*: 60–70% of maximum heart rate *Time*: 24 weeks *Type*: resistance, aerobic, balance, proprioception, stretching training *Settings*: department gym, patient’s room, or outdoors, outdoor camp CG: Placebo intervention	Retention: 97.6% Adherence: NR	No	**Functional capacity** (6 MWT) **Flexibility** (sit-and-reach test) **Balance** (flamingo balance test) **Upper limb strength** (1 kg medicine-ball launch) **Lower limb strength** (Myotest and chair test) **Trunk muscle endurance** (bridge trunk muscle endurance test) **Abdominal muscle endurance** (sit-up score) **Weight** **BMI** **Fat mass** (impedance meter) **Lean mass** (impedance meter) **Self-esteem** (PSI-VSF) **QoL** (VSP-A)	-↑6 MWT -↑Flexibility -↑Balance -↑Upper limb strength -↑Lower limb strength -↑Trunk muscle endurance -↑Abdominal muscle -↑Endurance -↑Self-esteem -↑QoL
Stossel, et al., 2020 [34]	*N* = 33 EG: (*n* = 16) CG: (*n* = 17)	EG: 10.6 ± 5.2 CG: 11.4 ± 4.3	Mixed cancer	During treatment	EG: *Frequency*: 3 days/week *Intensity*: 60~75% of estimated maximum heart rate *Time*: 6~8 weeks *Type*: resistance and aerobic training *Settings*: hospital CG: Usual care	Retention: 72.2% Adherence: NR	No serious adverse events	**Muscle strength** (handheld dynamometers) **Walking performance** (6 MWT) **BMI**. **Body composition** (phase angle) **Fatigue** (the PedsQL 3.0 Multidimensional Fatigue Scale) **PA** (the German MoMo questionnaire), **Hours out of bed** (Semi-Structured Interview), **HRQOL** (The German-language KINDL questionnaire)	-↑Leg strength -↑Walking performance -↓Fatigue -↑^a^ Self-esteem -↑^a^ Self-reported strength and endurance capacity

Abbreviations: 5RM, the five-repetition maximum; 6MWT, 6 min walking test; BMI, body mass index; CG, control group; CUHK-PARCY, the Chinese University of Hong Kong Physical Activity Rating for Children and Youth; DSDP, the Daily Sleep Diary-Parent; EG, experimental group (exercise group); EQ-5D-Y, the EuroQOL–youth version questionnaire; FS-A, The Fatigue Scale for 13 to 18 Year Olds; FS-C, The Fatigue Scale for 7 to 12 Year Olds; FS-P, The Fatigue Scale: Parent Version; FS-S, The Fatigue Scale: Staff Version; HRQOL, Health-Related Quality of Life; PA, physical activity; PASCQ, the Physical Activity Stages of Change Questionnaire; PA-SE, Physical Activity Self-Efficacy scale; PedsQL, the Pediatric Quality of Life Inventory; PSI-VSF, Physical Self-Inventory—Very Short Form; QALYs, quality-adjusted life years; QoL, quality of life TUG, Timed Up and Go; TUDS, Timed Up and Down Stairs; VSP-A, Vecu et Sante Percue de l’Adolescent; VO_2peak_, peak oxygen uptake. Additional information: ^a^ Self-esteem, self-reported strength and endurance capacity were measured with the subscales of HRQOL.

## Data Availability

Data are available in a publicly accessible repository.

## Appendix A

Search terms: (cancer OR oncology OR tumor OR tumour OR neoplasm OR leukemia OR leukaemia OR carcinoma OR sarcoma OR malignant OR maligna*) AND (pediatric OR paediatric OR child OR child* OR kid OR infant OR adolescent OR adoles* OR teenager OR teen*) AND (physical activity OR exercise OR aerobic OR resistance OR training OR sport OR physical therapy OR rehabilitation)

Filter: Humans; English; Child: birth-18 years

Article types: Randomized Controlled Trial

Search fields: All Fields

Database: PubMed

Result: 2220

Search terms: (cancer OR oncology OR tumor OR tumour OR neoplasm OR leukemia OR leukaemia OR carcinoma OR sarcoma OR malignant OR maligna*) AND (pediatric OR paediatric OR child OR child* OR kid OR infant OR adolescent OR adoles* OR teenager OR teen*) AND (physical activity OR exercise OR aerobic OR resistance OR training OR sport OR physical therapy OR rehabilitation)

Filter: Randomized Controlled Trial

Search fields: Title, Abstract, Author keywords

Database: Embase

Result: 314

Search terms: (cancer OR oncology OR tumor OR tumour OR neoplasm OR leukemia OR leukaemia OR carcinoma OR sarcoma OR malignant OR maligna*) AND (pediatric OR paediatric OR child OR child* OR kid OR infant OR adolescent OR adoles* OR teenager OR teen*) AND (physical activity OR exercise OR aerobic OR resistance OR training OR sport OR physical therapy OR rehabilitation)

Filter: Trials;

Source: CT.gov, ICTRP, CINAHL

Search fields: Title Abstract Keyword

Database: Cochrane Library

Result: 811

Search terms: (cancer OR oncology OR tumor OR tumour OR neoplasm OR leukemia OR leukaemia OR carcinoma OR sarcoma OR malignant OR maligna*) AND (pediatric OR paediatric OR child OR child* OR kid OR infant OR adolescent OR adoles* OR teenager OR teen*) AND (physical activity OR exercise OR aerobic OR resistance OR training OR sport OR physical therapy OR rehabilitation)

Filter: Articles; English

Research Areas: Oncology, Pediatrics, Hematology, Health Care Sciences Services, Nursing, Rehabilitation, Sport Sciences

Search fields: Topic

Database: Web of Science core collection

Citation indexes: Science Citation Index Expanded; Social Science Citation Index; Emerging Sources Citation Index

Result: 5438
